# Fear of negative appearance evaluation and attitude towards mammography: Moderating role of internal health locus of control,cancer worry and age

**DOI:** 10.34172/hpp.2020.35

**Published:** 2020-07-12

**Authors:** Saeed Pahlevan Sharif, Ashraf Sadat Ahadzadeh, Fon Sim Ong, Navaz Naghavi

**Affiliations:** ^1^Taylor’s Business School, Taylor’s University Lakeside Campus, No. 1 Jalan Taylors, 47500 Subang Jaya, Selangor, Malaysia; ^2^Department of Journalism, Xiamen University Malaysia Campus, Jalan Sunsuria, 43900 Sepang, Selangor, Malaysia; ^3^Nottingham University Business School, The University of Nottingham Malaysia Campus, Malaysia

**Keywords:** Fear of negative appearance evaluation, Attitude towards mammography, Breast neoplasms internal-external control, Breast neoplasms worry, Age factors

## Abstract

**Background:** Mammography screening tends to reduce mortality rate through early detection. One of the barriers to mammography screening is fear of negative appearance evaluation(FNAE). This study investigated the impact of internal health locus of control, breast cancer worries and age on the relationship between FNAE and attitude towards mammography.

**Methods:** A cross-sectional, questionnaire-based survey design was used. Samples were Iranian women, living in Iran, aged at least 30 years old, without any history of cancer, and had not performed mammography previously based on self-report. In total, 823 samples were collected through conducting an online survey from April to June 2016. The questionnaire consisted of several instruments including attitude toward breast cancer screening procedures scale, FNAEscale, the internal dimension of the multidimensional health locus of control, and two items to measure breast cancer worry. Using covariance-based structural equation modeling the model was tested.

**Results:** The interaction of FNAE with internal health locus of control (β = -0.128, P<0.05,CI: -0.200, -0.056), breast cancer worry (β = 0.090, P<0.05, CI: -0.162, -0.017), and age (β =-0.095, P<0.05, CI = -0.163, -0.026) was significant. The three tested moderators dampened the positive relationship between FNAE and negative attitude towards mammography.

**Conclusion:** More information about the screening procedure should be given to women to overcome their fear. The findings indicate the need for interventions seeking to shift women’s health locus of control from external to internal. Women with low level of cancer worry need more attention.

## Introduction

Breast cancer is a principal form of cancer and a prevalent malignancy among women^1^ accounting for 25% of all female cancer globally.^2^ In Iran specifically breast cancer is reported as the most common malignancy among women.^3^ Strong evidence shows that breast cancer screening and mammography screening tend to reduce mortality rate through early detection.^4-7^ This has attracted researchers to study the barriers to mammography screening.

While there is a growing body of research on a barriers such as lack of knowledge, fear of breast cancer diagnosis,^8,9^ possible pain experienced during mammography, low self-efficacy, lack of health motivation, lack of access to health-care, and lack of physician referral,^10-12^ research on the role of fear of negative evaluation on mammography screening is scarce. Due to its consequences,^13-15^ fear of negative evaluation would be an important detrimental factor in women’s mammography screening. Thus, this study attempts to examine if the more specific fear of negative appearance evaluation (FNAE) affect women’s attitude toward mammography screening.

Moreover, this study goes one step further and investigates the mechanism behind this relationship by introducing three moderators that may weaken or strengthen the relationship between FNAE and attitude towards mammography screening.

Controlling for country specific sociocultural influence, this study focuses on Iran women along three additional major reasons. First, body and physical appearance dissatisfaction is prevalence among Iranian women^16,17^ which leads to their lower self-esteem and strong desire for plastic surgery.^18^ Second, Iranian women have shown low tendency to undertake regular breast cancer and mammography screening.^10,19,20^ Third, breast cancer is the most prevalent chronic disease among Iranian women^20^ and the peak age of incidence is ten years earlier than developed countries.^21^

This research contributes to the growing interest among researchers in explaining the mechanism that shapes attitudes towards mammography screening by focusing on Iranian women who have no prior breast cancer screening experience. Therefore, to fill the gap in the knowledge, the current study aims to investigate the moderating role of internal health locus of control, breast cancer worries and age in the relationship between FNAE and attitude towards mammography among Iranian women without breast cancer screening experience.

### Theoretical framework

Fear of negative evaluation refers to people’s concern about the prospect of negative interpersonal evaluation, thus avoiding situations where they may be evaluated.^22,23^ Fear of negative evaluation has recently emerged as a contributor to negative behaviors and dejected feelings. Studies revealed that individuals with high levels of fear of negative evaluation tend to experience more psychological distress such as anxiety in social situations.^24,25^

In mammography screening, the FNAE may be one of the barriers to positive attitude towards mammography screening. The sense of dread associated with being evaluated unfavorably by others i.e. physicians, nurses and health providers may generate negative affective feelings towards mammography and prevent women from undergoing a mammography.^22^ In fact, women’s concern about others’ judgment may adversely affect their attitude towards mammography. Thus, the following hypothesis is developed:

H1: FNAE is negatively correlated with attitude towards mammography.

This study suggests that the relationship between FNAE and attitude towards mammography depends on other factors such as health locus of control. Health locus of control is defined as the extent to which individuals attribute their health to their own actions or to environmental circumstances and powerful external agents.^26^ An internal locus of control is characterized by the belief that positive health results from one’s own personal efforts. In contrast, an external locus of control suggests that health is due to the influence of fate, powerful others, or supernatural sources.^1^

If women believe that they are primarily in control of their health they are more likely to engage in screening behaviors, such as performing breast self-examination, clinical examination and mammography. A study found that adult women who practice breast self-examination tend to be less inclined to depend upon powerful others such as their health care provider.^27^ Internal locus of control was also found to be significantly associated with cervical cancer screening behavior in Nigerian women.^28^ Likewise, a study showed that women with lower internal health locus of control were two times more likely to have inadequate abnormal screening mammography follow-up than women with higher mean internal health locus of control.^29^ In contrast, a group of studies provided no support for internal health locus of control as a predictor of screening behavior. For example Holm et al^30^ did not find internal health locus of control to be a determinant of women’s mammography behavior. Similarly, the results of a cross-sectional study on a sample of African-American and Latino females showed that internal health locus of control and chance health locus of control were not significant predictors of up-to-date cervical cancer screening.^31^

Internal health locus of control may moderate the correlation between FNAE and mammography attitude. Women with a high level of internal health locus of control may more likely have a positive mammography attitude compared to women with lower internal health locus of control. Thus, it is expected that internal health locus of control weakens the negative relationship between FNAE and mammography attitude. In order to test this postulation, the following hypothesis is developed.

H2. Internal health locus of control moderates the negative relationship between FNAE and attitude towards mammography.

Cancer worry, defined as psychological perception of the risk of succumbing to cancer,^32^ is another factor proposed by this study as a moderator on the link between FNAE and attitude towards mammography. Cancer worry acts as a motivating factor for health-proactive behavior in some individuals and may drive them to undergo appropriate screening tests.^33^ Findings of previous empirical studies on the relationship between cancer worry and screening behavior are mixed. Some studies showed that high level of cancer worry results in screening behaviors including breast self-examination, mammography, and clinical breast examination.^34^ In some other studies, results showed that women who reported moderate levels of worry were also more likely to use mammography annually than those who were either mildly or severely worried.^35,36^ Cancer worry was also found to be a significant predictor of mammography adherence after controlling for the effect of prior utilization, feelings of vulnerability, general distress,^37^ education and socioeconomic status.^38^ Furthermore, there is evidence that breast cancer worry boosts screening only when some buffering factors such as self-efficacy come into play.^39^ However, the reverse may happen, that is, it may cause distress and screening avoidance.^40^

Hay et al^41^ in their meta-analysis of studies on cancer worry-screening link explained that the contradictory findings of the past studies could be due to their cross-sectional design and measuring cancer worry and screening adherence simultaneously. They concluded that breast cancer worry is an indubitably positive determinant of cancer screening behavior.

Based on the literature reviewed above, breast cancer worry may buffer the correlation between FNAE and mammography attitude. Women with higher levels of breast cancer worry may more likely have a positive mammography attitude such that breast cancer worry mitigates the negative relationship between FNAE and mammography attitude. Therefore:

H3. Breast cancer worry moderates the negative relationship between FNAE and attitude towards mammography.

Cancer screening behavior is also age dependent. Considering the fact that older women are more concerned with cancer risk^42^; have higher self-efficacy for cancer screenings^43,44^ and are more inclined to peer encouragement to engage in cancer screenings,^45^ they are more likely to show more positive attitude towards mammography regardless of others’ negative judgment about their body as compared to younger women. Thus, age may attenuate the relationship between FNAE and favorable attitude towards mammography. In other words, the association between FNAE and attitude towards mammography may be a function of women’s age, such that FNAE may be a weaker barrier to undertake mammography for older women. Thus, the following hypothesis is proposed:

H4. Age moderates the negative relationship between FNAE and attitude towards mammography.

## Materials and Methods

### Design and sample

A cross-sectional, questionnaire-based survey was used to test the hypotheses developed to examine the moderating role of internal health locus of control, breast cancer worry and age in the relationship between FNAE and attitude towards mammography among Iranian women with no previous experience of mammography screening. The samples are part of a broader project on Iranian women body image^16^ that were collected using convenience sampling method through online survey administered from April to June 2016. The survey link was posted in active social media groups in Iran with members from different rural and urban areas of the country. For the purpose of this study, using several inclusion criteria, a total of 823 Iranian women who participated in the main study were selected. More specifically, samples for this study were Iranian women, (1) living in Iran, (2) aged at least 30 years or older (3) who had not been diagnosed with any types of cancer, and (4) had not performed mammography previously based on self-report. The results of power analysis ^46^ and G*Power 3.1.7 based on a fixed model of linear multiple regression analysis showed that a total sample of 791 samples is enough to achieve an alpha less than or equal to 0.05 (two-tailed) and power greater than or equal to 80% with a small effect size of 0.02, critical *F* = 1.892.

### Instruments

A self-administered questionnaire was developed to obtain the necessary data. All scales were translated into Persian language using the forward-backward translation technique and following the World Health Organization protocol.^47^

*Attitudes toward mammography* was measured using the 14-item Attitude toward Breast Cancer Screening Procedures Scale (ABCSPS)^48^ that measures women’s negative attitudes and beliefs about different aspects of mammography, clinical breast examinations, and breast cancer (e.g. “I worry about the amount of radiation I would get when I have a mammogram”). The instrument showed good reliability and validity in different ethnic groups.^48,49^ The response was scored on a seven-point Likert scale ranging from 1 to 7. A higher score indicates a more negative attitude toward mammography. *FNAE* was measured using the 6-item Fear of Negative Appearance Evaluation Scale (FNAES) validated by Lundgren et al^50^ that addresses participants’ apprehension about negative appearance evaluation by others (e.g. “I am afraid other people will notice my physical flaws”). Items were scored on a six-point Likert scale ranging from 1 (not at all) to 6 (extremely). This study measured *Internal health locus of control* using the internal dimension of the 18-item Multidimensional Health Locus of Control (MHLC) scale, developed by Wallston et al.^51^ The internal dimension consists of six items and assesses an individual’s tendency to believe that health outcomes are due to his/her own behavior (e.g. “I am in control of my health”). Each item was measured using a seven-point Likert scale varying from 1 (strongly disagree) to 7 (strongly agree). To measure *Breast cancer worry* two items assessed frequency and influence on women’s daily life (i.e. “How often do you worry about developing breast cancer?” and “How much does worrying about developing breast cancer interfere with your everyday life?”). The scale was validated and used in previous studies.^52,53^ Each item was recorded on a seven-point Likert scale ranging from 1 (not at all) to 7 (very much). *Finally,* participants’ socio-demographic characteristics such as age, marriage status, education level, employment status, economic situation, insurance coverage, BMI, and cancer history in the family were captured in the questionnaire.

### Data analysis 

This study used IBM SPSS Statistics (version 20, IBM Corporation, Armonk, NY, USA) and AMOS (version 21, Chicago: IBM SPSS) software packages to test the research hypotheses. There are two approaches to conduct structural equation modeling (SEM) including covariance-based SEM (CB-SEM) and variance-based SEM (VB-SEM) also known as partial least squares-SEM. CB-SEM has become the more widespread method in research due to its advantages compared with PLS-SEM. This study used CB-SEM as it assesses the model as a whole using model fit indices and the model developed in this study consists of several latent reflective constructs (i.e. ABCSPS, FNAE, internal health locus of control, and breast cancer worry). CB-SEM is comprised of two steps including measurement model assessment and structural model assessment.^54^ Initially, a confirmatory factor analysis (CFA) was conducted and the model fit was assessed using model fit indices. Construct reliability, convergent validity, and discriminant validity of the constructs were assessed using Cronbach’s alpha, composite reliability (CR), average variance extracted (AVE), average shared squared variance (ASV), and maximum shared squared variance (MSV). Cronbach’s alpha and CR greater than 0.7 as well as significant factor loadings indicated good reliability and convergent validity. To establish discriminant validity, ASV and MSV should be less than their respective AVE.^55^ Next, using imputation method, the constructs were replaced with their latent variable score. The structural model was developed, and hypotheses were tested performing bootstrapping with 2000 replications. Bootstrapping is a nonparametric distribution-free technique that does not assume anything about the underlying distribution. Conditional relationship between FNAE and attitude toward mammography at different values of the moderators were computed and reported as well. The results of model testing were controlled for the effect of education level and insurance coverage. All tests were two-tailed and a *P* value of less than 0.05 was considered to be statistically significant.

## Results

The mean age of the participants was 40.06 (SD = 8.83). The sample was mainly from urban areas (95.5%), married (80.7%), unemployed (59.0%), and had diploma and above qualification (78.8%) and insurance coverage (84.9%). In terms of the reported BMI, 307 participants (37.3%) fell into normal weight, followed by 290 (35.2%) in underweight, 163 (19.81%) in overweight, and 63 participants (7.7%) into the obese group. Table 1 reports a summary of the participants’ socio-demographic characteristics.

Next, the factor structure was developed and validated by performing CFA. By following the modification indices, four pairs of the items’ measurement errors of ABCSPS were allowed to freely covary. Moreover, four items of ABCSPS and two items of internal health locus of control were deleted as they loaded weakly on their respective constructs. The revised measurement model had a good fit (*χ*^2^(199) = 585.763, *P* < 0.001, *χ*^2^/*df* = 2.944, goodness-of-fit index (GFI) = 0.939, comparative fit index (CFI) = 0.951, incremental fit index (IFI) = 0.951, normed fit index (NFI) = 0.927, standardized root mean square residual (SRMR) = 0.036, and root mean square error of approximation (RMSEA) (90% confidence interval (CI)) = 0.049 (0.044–0.053)). All item loadings were greater than 0.5 and significant (z-value between 9.941 and 28.296). Table 2 reports the results of the assessment of the constructs. The CR and Cronbach’s alpha of all constructs was greater than 0.7 indicating a good construct reliability and convergent validity.^55^ This study also estimated AVE of the constructs. AVE of FNAES (0.477) and ABCSPS (0.454) were slightly less than 0.5. However, according to Malhotra and Dash^56^ “AVE is a more conservative measure than CR. On the basis of CR alone, the researcher may conclude that the convergent validity of the construct is adequate, even though more than 50% of the variance is due to error.” (p. 702). Moreover, AVE of each construct was greater than its ASV and MSV establishing discriminant validity of all constructs.^57^

The results after controlling for the effect of education level and insurance coverage are reported in Table 3. There was a significant positive relationship between FNAES and ABCSPS that was used to measure negative attitudes towards mammography (*β* = 0.144, *P* < 0.001) providing support for H1. As ABCSPS is a measure of negative attitudes towards mammography, the significant positive relationship supported that FNAE is a barrier to take mammography, and therefore the relationship between FNAE and attitude towards mammography screening is negative and significant.

**Figure 1:**
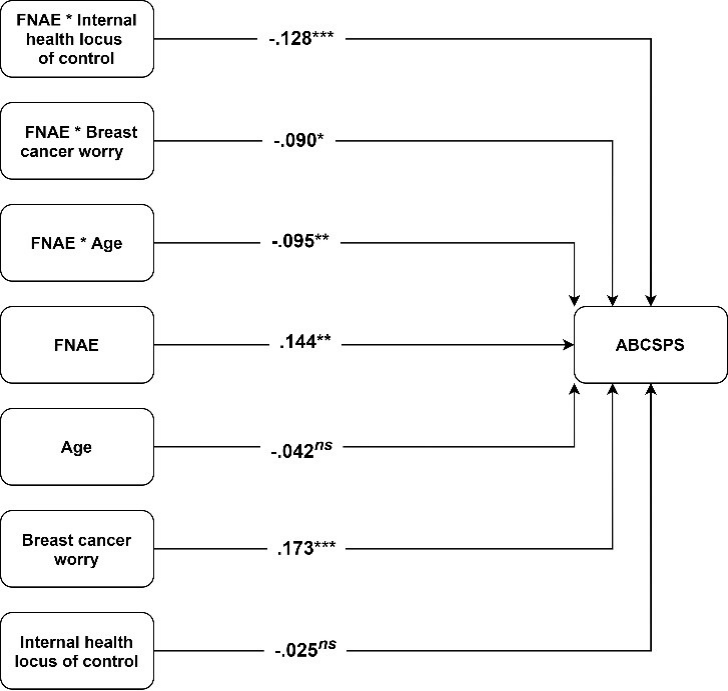
Structural model assessment results. Abbreviations: ABCSPS: Attitude toward breast cancer screening procedures scale, FNAE: Fear of negative appearance evaluation.

**Figure 2:**
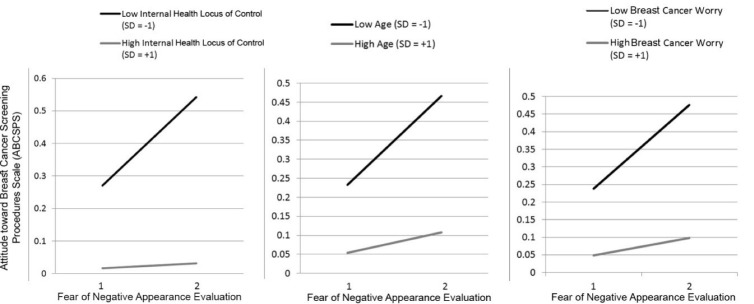
The conditional relationship between FNAE and ABCSPS. *P* values for the interaction of FNAE and Internal health locus of control, FNAE and Breast cancer worry, and FNAE and Age are 0.000, 0.015 and 0.007 respectively.

Also, the interaction of FNAES with internal health locus of control (*β* = -0.128, *P* < 0.05), breast cancer worry (*β* = -0.090, *P* < 0.05), and age (*β* = -0.095, *P* < 0.05) was significant which provided support for H2, H3, and H4 respectively. More specifically, the results indicated that the three moderators tested in this research dampened the positive relationship between FNAES and ABCSPS (negative attitudes towards mammography). Figure 1 shows the results of the assessment of the structural model. The conditional relationship between FNAES and ABCSPS showed that for the low (SD = -1) and medium (SD = 0) levels of the moderators, the relationship between FNAES and ABCSPS is positive and significant. However, for higher levels of the moderators (SD = +1), this study could not find any significant relationship between FNAES and ABCSPS. Figure 2 shows the relationship between FNAES and ABCSPS as a measure of negative attitudes towards mammography for low and high values of the moderators. The model explained 10% of the variance of attitudes towards mammography.

## Discussion and Conclusion

This study attempts to fill the gap in the literature related to the association between FNAE and women’s attitudes towards mammography screening. The moderating effects of internal health locus of control, breast cancer worry, and age on this relationship were tested.

The results showed a positive relationship between FNAES and ABCSPS scale which measures negative attitude towards mammography. The findings provide evidence that women’s FNAE by others can act as a barrier for mammography screening. This may be due to the fact that mammography screening requires physical examination of their body and exposure of their body to a medical professional. These results provide empirical evidence for the conceptual framework developed by Ridolfi and Crowther.^45^ They stated that “women experiencing body shame may avoid cancer screenings performed by a physician for fear that they will be negatively evaluated on the basis of the perceived flaws in their physical appearance” (p. 154). In this study, women with no past mammography screening experience showed a strong relationship between FNAE and attitude towards mammography screening. This corroborated with past studies on the association between body image and screening behavior.^58-61^ The results of a study by Chait et al^58^ on 93 women indicated that those with higher satisfaction with overall appearance and evaluating themselves as attractive reported more frequent skin self-examination. Clark et al^60^ showed that body image concern was one of the barriers to perform cancer screening for both men and women.

This study also provided support for the moderating effect of internal health locus of control, breast cancer worry, and age on the relationship between FNAE and attitudes towards mammography. More specifically, internal health locus of control, breast cancer worry, and age weakened the FNAE-attitudes towards mammography link. The findings show that FNAE could more likely act as a barrier towards positive mammography behavior in women who were less likely to have a sense of control over their health status, who were less worried about the prospect of contracting the disease, and who were younger.

Women who have a disposition toward internal health locus of control are more likely to actively participate in their own health care and health-related information seeking and as a result, they are more likely to have higher levels of awareness and self-efficacy for cancer screening which in turn may improve their attitudes towards cancer screening and mammography.^1,62^ Moreover, the findings lend support to the notion that being worried about cancer may have benefits, having a positive effect on women’s attitude towards cancer screening and prompting them to engage in screening behaviors such as mammography.^37,41^ Furthermore, due to the prevalence of cancer in older women,^42^ women who were older tend to have higher awareness and self-efficacy for breast cancer screenings and mammography. Also, they may be more susceptible to more peer and medical professional encouragement to undertake mammography. These may buffer the negative effect of the FNAE on their screening attitudes and as a result they may be more likely to undertake mammography regardless of their FNAE.^45^

The moderating effect of internal health locus of control, cancer worry, and age adds to our understanding of the relationship between FNAE and mammography screening behavior among women who have not had any prior mammography screening experience. This is a major contribution and it is useful for health policy and practices strategies that are aimed at early detection of breast cancer among women.

### Implications

The association between FNAE and attitudes towards mammography may inform the practices of health professionals who interact with female patients. To reduce the consequences of FNAE during mammography, health practitioners should provide reassurance that all health professionals are professionally trained to perform screening and they are bound by their code of ethics about patient care. More information about the screening procedure should be given to women to overcome their fear. Women may respond more positively to medical professionals’ advice when the recommendations are on their health instead of losing weight, and physical appearance.^45^ More importantly, women must be empowered to have more self-esteem. Indeed, communication approaches should avoid the fear appeal (whether it is for the disease or appearance shame), rather the message should be towards acceptance of individuals (e.g. all are beautiful) as equal beings and that dignity is preserved and upheld during mammography procedure.

The results of testing the moderating variables in the model produced useful implications. The stronger detrimental effect of FNAE on mammography attitude and behavior in younger women suggests paying special attention to this group of women. Communication message should highlight that cancer does not discriminate in terms of age and therefore the benefits of early detection apply to women of all ages. The findings indicate the need for interventions seeking to shift women’s health locus of control from external to internal in order to improve their attitudes towards mammography and cancer screening behaviors. Indeed, for women who are concerned about others’ appearance evaluation, internal locus of control can act as an effective buffering factor against the negative consequences of FNAE on women’s screening attitude and behaviors.

Women with low level of cancer worry need a more focused strategy, in particular for those who have a high level of FNAE. For example, choice of screening methods could be used in stages where they could be first encouraged to use self-examination method followed by mammography screening. Communications on mammography could emphasize on the benefits of screening that promote confidence among women who are in control of their health since health is one of the important factors that promotes subjective well-being.

### Limitations and recommendations for future studies

This study is not without limitations. Using an online survey and relying on self-report of experience of mammography would limit the generalizability of the findings. Also, considering the power of the test, the generalizability of the results and any interpretation should be done discreetly. Moreover, the majority of the participants were from urban areas which does not allow us to generalize the findings to the entire nation. Future studies should include samples from rural areas for representativeness as geographic location may affect screening behavior due to informational barriers or availability of resources. Moreover, the cross-sectional design of this study limits the causal conclusions that can be drawn from the results. Similar study could also be replicated in countries with different cultures to understand if the relationship between fear of negative evaluation and cancer screening attitude and behavior is culture specific. This study was not able to test the cultural sensitivity of women in mammography screening as data were collected in one single country with homogeneous population. Future studies should focus on cultural and sub-cultural influences on breast cancer screening. In particular, it is worth exploring body shame and body image disturbance in place of FNAE. Certainly, more work is needed to consider other variables such as personality traits, self-determination theory, and social acceptance that influence human behavior.

## Ethical approval

The study protocol and the consent form were approved by the ethical committee of the first author’s institution. All participants provided informed consent. The purpose of the study was explained to the participants and they were assured that all responses are anonymous, and participation in this study is voluntary. The authors assert that all procedures contributing to this work comply with the ethical standards of the relevant national and institutional committees on human experimentation and with the Helsinki Declaration of 1975, as revised in 2008.

## Competing interests

The authors declared no potential conflicts of interest with respect to the research, authorship, and/or publication of this article.

## Funding

This study was funded by an extension of Taylor’s University Research Grant (TRGS/ERFS/1/2015/TBS/014).

## Authors’ contributions

SPS involved in the conceptualization of the study, data collection, methods, results, and discussion. ASA and FSO involved in the introduction section. NN involved in the discussion. FSO and NN involved in editing the work..

## Acknowledgments

We thank all participants, Mastoureh Afshari, Khadijeh Irani Tehrani, Nahal Naghavi, and Fatemeh Irani Tehrani for their support.

**Table 1:** A summary of participants’ socio-demographic characteristics

**Characteristics**	**No.**	**%**
Marriage status		
Single	114	13.85
Married	664	80.68
Widow/divorced	45	5.47
Education level		
Primary school and below	87	10.57
Secondary school	87	10.57
Diploma	215	26.12
College	63	7.65
Degree	252	30.62
Masters/PhD	119	14.46
Living area		
Urban	786	95.50
Rural	37	4.50
Economy condition		
Very weak	12	1.46
Weak	57	6.93
Average	481	58.44
Good	254	30.86
Excellent	19	2.31
Employment status		
Full time	182	22.11
Part time	145	17.62
Unemployed	486	59.05
Retired	10	1.22
BMI category		
Underweight (BMI <18.5)	290	35.24
Optimal (18.5 < BMI <25)	307	37.30
Overweight (25 < BMI < 30)	163	19.81
Obese (BMI > 30)	63	7.65
Medical insurance coverage		
Yes	699	84.93
No	124	15.07

**Table 2:** Measurement model assessment

**Construct/measure**	**Factor loading**	**Cronbach's alpha**	**Construct reliability**	**Average variance extracted**	**Maximum shared square variance**	**Average shared squared variance**
ABCSPS		0.893	0.891	0.454	0.033	0.023
ABCSPS.3	0.654					
ABCSPS.4	0.666					
ABCSPS.5	0.505					
ABCSPS.6	0.731					
ABCSPS.7	0.746					
ABCSPS.8	0.571					
ABCSPS.9	0.782					
ABCSPS.10	0.763					
ABCSPS.11	0.636					
ABCSPS.13	0.633					
FNAES		0.916	0.883	0.563	0.152	0.067
FNAES.1	0.572					
FNAES.2	0.579					
FNAES.3	0.814					
FNAES.4	0.855					
FNAES.5	0.806					
FNAES.6	0.820					
IHLoC		0.779	0.784	0.477	0.016	0.009
IHLoC.12	0.657					
IHLoC.13	0.782					
IHLoC.17	0.694					
IHLoC.6	0.619					
BCW		0.776	0.774	0.633	0.152	0.064
BCW.1	0.724					
BCW.2	0.861					

Abbreviations: ABCSPS, Attitude toward breast cancer screening procedures scale, FNAES, Fear of negative appearance evaluation scale, IHLoC, Internal health locus of control, BCW, Breast Cancer worry.

**Table 3:** Structural model assessment (The effect of exogenous variables on ABCSPS)

**Exogenous variables**	**Standardized path coefficients**	**95% confidence level**		
		**Lower bound**	**Upper bound**	**P value**
FNAE	0.144	0.063	0.225	0.001
Internal health locus of control	-0.025	-0.093	0.044	0.484
Breast cancer worry	0.173	0.097	0.249	0.000
Age	-0.042	-0.116	0.033	0.273
FNAE * Internal health locus of control	-0.128	-0.200	-0.056	0.000
FNAE * Breast cancer worry	-0.090	-0.162	-0.017	0.015
FNAE * Age	-0.095	-0.163	-0.026	0.007
Insurance	-0.082	-0.147	-0.016	0.015
Education level	-0.036	-0.105	0.032	0.296
FNAE | Internal health locus of control = -1 SD	0.271	0.162	0.381	0.000
FNAE | Internal health locus of control = +1 SD	0.016	-0.091	0.123	0.772
FNAE | Breast cancer worry = -1 SD	0.233	0.111	0.356	0.000
FNAE | Breast cancer worry = +1 SD	0.054	-0.039	0.147	0.256
FNAE | Age = -1 SD	0.238	0.140	0.337	0.000
FNAE | Age = +1 SD	0.049	-0.064	0.162	0.395

Abbreviation: FNAES, Fear of negative appearance evaluation.
